# Synthesis and properties of multi-functionalized graphene quantum dots with tunable photoluminescence and hydrophobicity from asphaltene and its oxidized and reduced derivatives†

**DOI:** 10.1039/d2na00445c

**Published:** 2022-08-22

**Authors:** Maryam Aghajamali, Mariana Arpini Vieira, Razieh Firouzi-Haji, Kai Cui, Jae-Young Cho, Adam Johan Bergren, Hassan Hassanzadeh, Alkiviathes Meldrum

**Affiliations:** Department of Chemical & Petroleum Engineering, Schulich School of Engineering, University of Calgary Calgary AB T2N 1N4 Canada maryam.aghajamali@ucalgary.ca hhassanz@ucalgary.ca; Department of Physics, University of Alberta Edmonton AB T6G 2E1 Canada ameldrum@ualberta.ca; Nanotechnology Research Centre, National Research Council of Canada Edmonton AB T6G 2M9 Canada adam.bergren@nrc.ca; Department of Chemistry, University of British Columbia Kelowna BC V1V 1V7 Canada

## Abstract

Graphene quantum dots (GQDs) with tunable photoluminescence (PL) and hydrophobicity were synthesized from an abundant natural carbon source containing nitrogen, sulfur, and oxygen heteroatoms. Asphaltene and its oxidized and reduced derivatives were used as precursors to produce GQDs in organic solvents (*i.e.*, methanol, toluene, and chloroform) using a facile ultrasonication technique. Asphaltene surface chemistry was tuned by sequential oxidation and reduction to investigate the surface effects on GQD properties. Spectroscopic characterizations confirmed the presence of N, S, and O heteroatoms and different electron-donating and electron-withdrawing groups. Microscopic characterizations revealed that these crystalline carbon nanomaterials have mono-layered or multi-layered structures with lateral sizes in the range of ∼5–15 nm. The asphaltene-derived GQDs exhibit tunable PL with emission colors ranging from blue to orange, depending on the carbon precursor and the organic solvent. Solvent exchange studies also revealed that asphaltene and its derivatives contain hydrophilic and hydrophobic fractions, resulting in varied hydrophobicity of the synthesized GQDs. Adding to the appeal of the present work, PL quenching of GQD-silica hybrid materials upon exposure to nitro-aromatics confirms that these GQDs can be incorporated to different host materials for advanced sensing or optoelectronic applications.

## Abstract 2

Graphene quantum dots with tunable photoluminescence and hydrophobicity were synthesized from an abundant natural carbon source containing nitrogen, sulfur, and oxygen heteroatoms.

## Graphical abstract

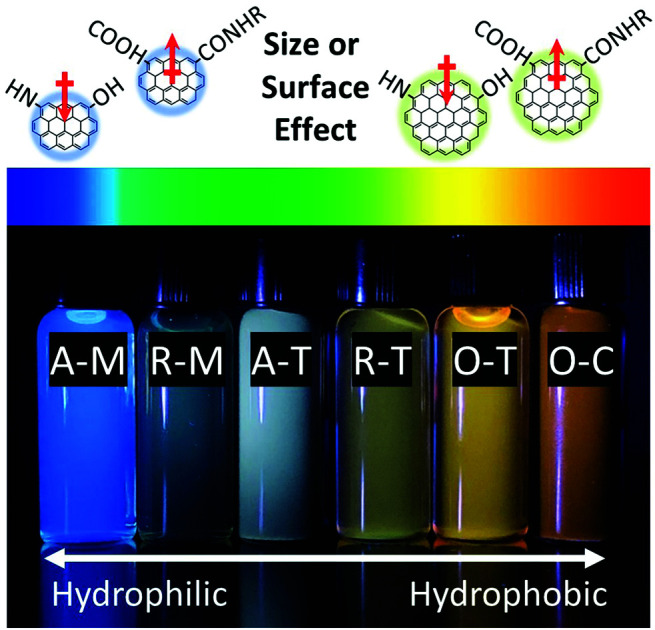

## 1. Introduction

As the newest member of the carbon nanomaterial family, graphene quantum dots (GQDs) offer attractive properties such as stable photoluminescence (PL), a tunable band gap dependent on quantum confinement or edge effects, ease of surface functionalization, low toxicity, and potentially low preparation cost.^1–4^ Some prototype applications that could benefit from these characteristics have been shown to include sensing,^4–7^ bioimaging,^8–11^ photocatalysis,^12–15^ electrocatalysis,^16–19^ optoelectronic,^20–23^ and energy storage and conversion systems.^24–28^

GQDs are promising substitutes for traditional quantum dots containing toxic heavy metals or expensive rare elements, while preserving many of their unique properties. GQDs are generally thought of as graphene nanomaterials having fewer than 10 graphene layers and lateral dimensions smaller than 100 nm.^1,2^ They can be synthesized from various carbon precursors using bottom-up or top-down approaches.^2,3^ Top-down methods involve the breaking down of larger particles and require methods such as oxidative cleavage,^29^ reductive cleavage,^30^ or physical cleavage using ultrasonication.^31^ These methods use cost-effective bulk carbon materials as precursors and are considered for large-scale GQD applications.

Graphite and its derivatives are the most common precursors for the synthesis of GQDs in top-down methods.^2,3^ These carbon precursors are relatively expensive and may require multiple steps involving a mixture of highly concentrated acids, strong oxidizing agents, or high temperatures (∼200 °C) to produce GQDs.^2,3^ Moreover, GQDs obtained from graphite and its derivatives have many limitations that restrict their applications. Some of these issues could be addressed by the surface functionalization of GQDs with single or multiple heteroatoms (*e.g.*, nitrogen, sulfur) that can modify their properties,^2,3^ but this requires additional post-synthesis steps. Furthermore, most synthesized GQDs are hydrophilic due to the presence of abundant oxygen-containing functional groups on their surface. This limits their potential sensing,^32^ optoelectronic,^33^ and energy storage applications^34^ since routine fabrication processes require the materials to be soluble or dispersible in organic solvents. Therefore, new precursors and strategies are needed to produce GQDs with improved properties for diverse large-scale applications.

Petroleum materials such as coal,^35^ petroleum coke,^36^ and petroleum asphaltene^37^ have received attention for GQD syntheses because they are low-cost and abundant. Among these precursors, asphaltenes are a promising candidate for the synthesis of GQDs because of the structural similarities between asphaltenes and the GQD core, which can facilitate the straightforward conversion of asphaltenes into GQDs. Asphaltenes are composed of a polycyclic aromatic carbon core (∼4–10 aromatic rings) containing different heteroatoms (*i.e.*, nitrogen, sulfur, and oxygen) and functional groups surrounded by short alkyl chains.^38–40^ Therefore, they can be considered as a natural carbon source for the synthesis of multi-functionalized GQDs. These properties could sidestep the additional steps needed to synthesize N- and/or S-functionalized GQDs with improved optical properties.^41–44^

Apart from their interesting chemical structure, asphaltenes are a low-cost byproduct of the oil refining process, which makes them attractive for the mass production of GQDs. Surprisingly, despite their unique chemical and structural aspects, there are only a few reports on the synthesis of GQDs from petroleum asphaltene.^37,45,46^ Early studies focused on only green-fluorescent water-soluble GQDs obtained by chemical oxidation in mixed acids.^37,45,46^ However, surface chemistry and solvent effects on the optical properties of asphaltene-derived GQDs were not explored. Moreover, there are no reports on asphaltene-derived GQDs with tunable PL and hydrophobicity, which could enable a range of sensing, optoelectronic, and energy storage and conversion applications.

In this work, we synthesized multi-functionalized GQDs with tunable properties from asphaltene and its derivatives using a facile ultrasonication technique. The carbon precursors included petroleum asphaltene and its oxidized and reduced products, from which functionalized GQDs were synthesized in organic solvents with different polarity and hydrophobicity (*i.e.*, methanol, chloroform, and toluene). The asphaltene surface was modified by sequential oxidation and reduction, allowing a more comprehensive investigation of the role of surface chemistry on the GQD optical properties. Additionally, the solvent effect on GQD properties was explored by tuning the hydrophobicity and polarity of the organic solvent.

## 2. Experimental section

### 2.1. Materials

All reagents were used as received unless otherwise stated. Water- and sand-free Athabasca bitumen was provided by an oil company in Alberta, Canada. Nitric acid (Certified ACS Plus) was obtained from Fisher Chemical. Hydrazine monohydrate (98+%) was purchased from Alfa Aesar. Heptane (reagent grade), toluene (HPLC grade), methanol (HPLC grade), chloroform (HPLC grade), and tetrahydrofuran (HPLC grade) were purchased from BDH VWR Analytical. Nylon and PTFE syringe filters (0.2 μm and 25 mm) with polypropylene housing were purchased from VWR International.

### 2.2. Separation of asphaltene from bitumen

Asphaltene (ASP) was separated from Athabasca bitumen using heptane following a procedure described elsewhere.^47^ Briefly, a specific amount of bitumen was mixed with heptane at the ratio of 1 g of bitumen per 40 mL of the solvent. The mixture was then sonicated for 50 min using an ultrasonic bath (VWR Ultrasonic Cleaner) and allowed to settle for 24 h. After one day, about 75 vol% of the mixture was filtered using Whatman® filter paper #2 (Note: do not shake the mixture before filtration). Next, the fresh solvent was added to the remaining mixture at about 10 vol% of the initial solvent amount, sonicated for 45 min, and allowed to settle for 15 h. The mixture was then filtered using the previous filter paper, and the asphaltene precipitate was washed with fresh heptane until the liquid leaving the filter paper was almost colorless. The separated asphaltene, after drying in a vacuum oven at room temperature, was ground using a mortar and pestle and stored in a standard glass vial.

### 2.3. Oxidation of asphaltene

Asphaltene powder (2.0 g) was mixed with HNO_3_ (7 M, 100 mL) in a single-neck round-bottom flask and oxidized at 75 °C overnight (**Caution!** HNO_3_ is corrosive and must be handled with extreme care). The resulting suspension was cooled to room temperature, and the particles were separated by vacuum filtration and rinsed with de-ionized (DI) water until the liquid leaving the filter was neutral. The asphaltene oxide (AO) product was dried in a vacuum oven at 75 °C overnight, ground using a mortar and pestle, and stored in a standard glass vial.

### 2.4. Reduction of asphaltene oxide

Asphaltene oxide powder (1.0 g) was dispersed in DI water (100 mL) in a single-neck round-bottom flask and sonicated for three hours using an ultrasonic bath (VWR Ultrasonic Cleaner). Hydrazine monohydrate (1.0 g) was added to the AO suspension and stirred at 80 °C overnight to produce reduced asphaltene oxide (RAO). After cooling to room temperature, the resulting particles were separated by vacuum filtration and washed with DI water. The RAO product was ground using a mortar and pestle and stored in a standard glass vial after drying in a vacuum oven at room temperature overnight.

### 2.5. Synthesis of Graphene Quantum Dots (GQDs)

GQDs were synthesized from ASP, AO, and RAO precursors using a facile ultrasonication technique. ASP, AO, and RAO powders (100 mg) were dispersed in 5 mL organic solvents (*i.e.*, methanol, chloroform, and toluene) in glass vials (20 mL). All dispersions were sonicated for three hours using an ultrasonic bath (VWR Ultrasonic Cleaner), and the GQDs were extracted into the organic solvents. The supernatant from each vial was separated after the settlement of unreacted precursor materials, filtered through 0.2 μm Nylon or PTFE syringe filters (Nylon for methanol samples; PTFE for chloroform and toluene samples), and stored in standard glass vials. The chemical yield of GQDs depends on the solubility of their carbon source in the organic solvent, and it can be increased by multiple solvent addition and extraction.

### 2.6. GQD-silica hybrid materials and PL quenching studies

GQD-free silica alcogels were prepared *via* a base-catalyzed sol–gel processing following a literature procedure.^48^ To prepare GQD-silica hybrid materials, silica alcogels were immersed in GQD solutions (1 mg mL^−1^) for two days. After two days, the GQD solution was removed from each vial, leaving only the gel. For PL quenching studies in the gel-GQD hybrid materials, 5 mL of an aqueous solution of 4-nitrophenol (10^−6^ M) was added to each vial and photographs of the gels were then recorded with a Sony a6400 consumer camera.

### 2.7. Materials characterization

Precursor powders and GQD solutions were used for characterizations unless otherwise indicated. Fourier transform infrared (FTIR) spectra of all materials were obtained using a Shimadzu IRTracer-100 FTIR spectrometer. The FTIR samples were prepared by drop-coating solutions of precursors and GQDs in tetrahydrofuran onto an IR transparent window (*e.g.*, ZnSe window). Carbon, hydrogen, nitrogen, and sulfur (CHNS) contents were measured using a Thermo Scientific Flash 2000 Organic Elemental Analyzer equipped with Eager Xperience software. Raman spectra were acquired using a WiTec Alpha 300 confocal Raman microscope equipped with a 532 nm excitation laser. X-ray diffraction (XRD) patterns were recorded using a Rigaku MultiFlex diffractometer with a Cu-Kα X-ray source.

X-ray photoelectron spectroscopy (XPS) was performed using a Kratos AXIS 165 instrument. The base pressure and operating chamber pressure were maintained below 10^−9^ Torr. A monochromatic Al Kα source (*hν* = 1486.6 eV) was used at 15 mA and 14 kV to irradiate the samples. Survey scans spanned from the binding energy of 1100 to 0 eV, collecting spectra with a pass energy of 160 eV and a step of 0.3 eV. For high-resolution spectra, the pass energy and the step were 20 and 0.1 eV, respectively, with a dwell time of 200 ms. Charge neutralization was applied to stabilize spectra during spectra collection. CasaXPS software (VAMAS) was used to interpret spectra. All spectra were internally calibrated to the C 1s emission (284.8 eV). After calibration, the background was subtracted using a Shirley-type background to remove most of the extrinsic loss structure. The high-resolution S 2p region was fitted to S 2p_3/2_ and S 2p_1/2_ components, with spin–orbit splitting fixed at 1.18 eV, and the S 2p_3/2_/S 2p_1/2_ intensity ratio set to 2/1.

Transmission electron microscopy (TEM) and high-resolution (HR) TEM images were obtained using a Hitachi-9500 electron microscope equipped with a LaB_6_ filament and operated at an accelerating voltage of 100 kV. The TEM samples were prepared by drop-coating dilute GQD solutions onto an ultra-thin continuous carbon film grid or by casting the powdered sample onto the grid (for O–C and R–C samples). Particle size distributions were measured manually by counting at least 200 particles using ImageJ software (1.48v).

For AFM measurements, clean HOPG (highly ordered pyrolytic graphite) substrates were prepared to deposit the samples by spin-coating at 2500 rpm for 30 s to have well-dispersed GQDs. The sample surface was observed using a Digital Instruments/Veeco Instruments MultiMode Nanoscope IV AFM equipped with an E-scanner. To acquire optimized a height profile, high-resolution (Rc < 1 nm) silicon cantilevers (MikroMasch USA, Inc.) with low spring constants of 4.5 N m^−1^ were used in tapping mode (TM-AFM). To obtain a clear image from the surface, a low scan rate (0.5–1 Hz) and amplitude setpoint (1 V) were chosen during measurement.

UV-Vis absorption spectra were collected using a PerkinElmer Lambda 1050 UV-Vis spectrophotometer. PL spectra of all GQDs in methanol, chloroform, and toluene were obtained using the 351–364 nm lines of an Ar+ ion laser as the excitation source. For these measurements, the samples were placed in 1 cm quartz cuvettes. The PL was focused by a double convex 5 cm focal length lens, collected by optic fiber, passed through a 375 nm long-pass filter to eliminate scattered light from the excitation source, and fed into an Ocean Optics USB2000 spectrometer. The spectral response was calibrated by a blackbody radiator (Ocean Optics LS1). PL decay traces were taken using an Alphalas picosecond pulsed laser at a wavelength of 405 nm and a nominal pulse length of 40 ps. The PL was collected using a fiber optic system, passed through a long-pass filter to remove scattered excitation light, and directed into a Becker-Hickl HPM-100-50 photomultiplier tube with a response time of ∼100 ps. The decay traces were obtained using the SPC-130 module from Becker-Hickl.

## 3. Results and discussion

**Fig. 1:**
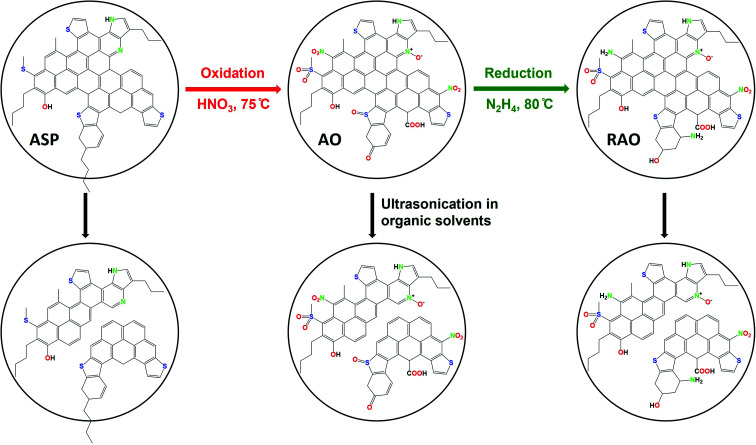
Synthesis of graphene quantum dots from asphaltene (ASP), asphaltene oxide (AO), and reduced asphaltene oxide (RAO).

The overall procedure for the synthesis of asphaltene-derived GQDs is shown in Fig. 1. While the details of the synthesis are reported in the Experimental section, we briefly reiterate them here for clarity. Asphaltene (ASP) was separated from bitumen using *n*-heptane.^47^ ASP was oxidized under a relatively mild oxidation condition using 7 M HNO_3_, to produce asphaltene oxide (AO). Reduced asphaltene oxide (RAO) was obtained by the reduction of AO with hydrazine. Luminescent N, S, and O-functionalized GQDs were synthesized from ASP, AO, and RAO carbon precursors in organic solvents (*i.e.*, methanol, toluene, and chloroform) *via* a facile and scalable ultrasonication method. Fig. 1 shows ASP oxidation to produce AO and its subsequent reduction to produce RAO. ASP and its oxidized and reduced derivatives were used as carbon precursors to prepare GQDs in organic solvents using an ultrasonication method.

### 3.1. Asphaltene and its oxidized and reduced derivatives

**Fig. 2:**
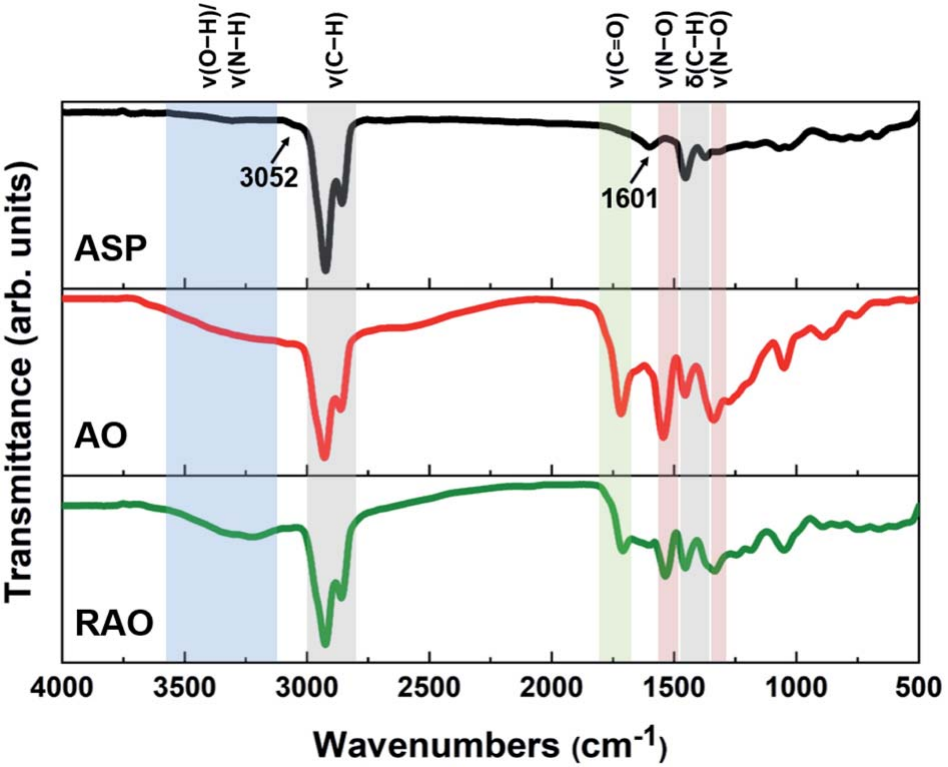
FTIR spectra of asphaltene (ASP) and its oxidized (AO) and reduced (RAO) derivatives.

The ASP, AO, and RAO precursors were characterized using Fourier transform infrared (FTIR) spectroscopy, X-ray photoelectron spectroscopy (XPS), Raman spectroscopy, X-ray diffraction (XRD), and elemental analysis. FTIR and XPS provide insight into the surface chemistry and composition of ASP, AO, and RAO precursors. Representative FTIR spectra show the expected features corresponding to the target surface groups (Fig. 2). All materials have strong absorptions at 2850–2950 and 1365–1475 cm^−1^ corresponding to C–H stretching and bending vibrations, respectively. The aromatic C=C and C–H stretching vibrations at ∼1601 and ∼3052 cm^−1^, respectively, are also visible in the FTIR spectrum of ASP.^49^ After oxidation of ASP with HNO_3_, a sharp absorption related to C=O stretching at 1700–1715 cm^−1^ and a broad absorption associated with O–H stretching at ∼3450 cm^−1^ appeared in the FTIR spectrum of AO. Asymmetric and symmetric N–O stretching vibrations at ∼1530 and ∼1340 cm^−1^, respectively, also become apparent after oxidation.^50^ Following the subsequent reduction with hydrazine to form RAO, we expected to observe a decrease in the intensity of peaks associated with oxygen-containing functional groups. The intensity of C=O stretching absorption decreased, while the intensity of O–H stretching absorption didn't decrease, and this peak became broader. The broad peak at ∼3200–3500 cm^−1^ might be due to the appearance of N–H stretching vibration at ∼3250 cm^−1^ that overlaps with the O–H stretching in the FTIR spectrum of RAO.

**Fig. 3:**
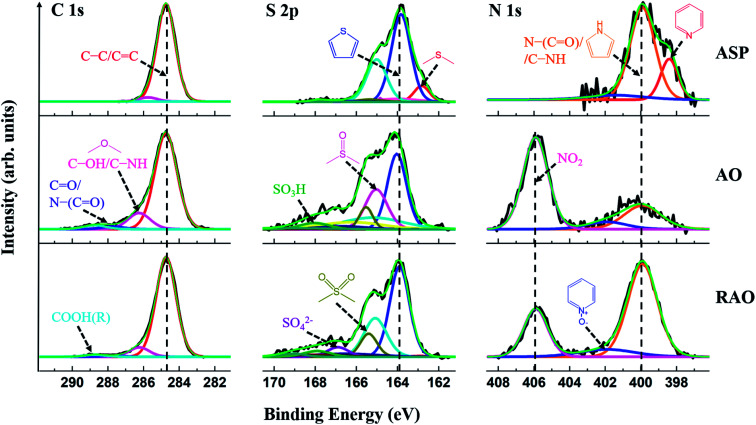
High-resolution XPS data for the C 1s, S 2p, and N 1s regions of ASP, AO, and RAO.

High-resolution XP spectra of the C 1s, S 2p, and N 1s regions of ASP, AO, and RAO precursors are shown in Fig. 3. The C 1s regions show graphitic carbon (C–C/C=C; ∼284.8 eV) as well as alcohol, ether, amine, ketone, amide, carboxylic acid, and/or ester carbon.^51^ The S 2p regions indicate aliphatic sulfur (2p_3/2_: ∼163.0 eV), thiophenic sulfur (2p_3/2_: ∼164.0 eV), sulfoxide, sulfone, sulfate, and/or sulfonic acid groups. The N 1s regions show pyridinic nitrogen (∼398.0 eV), pyrrolic nitrogen (∼400.0 eV), pyridine N-oxide, and/or nitro functionalities as well as amine and amide groups. Higher-binding-energy features in these regions are attributed to the oxidized species, which indicate that AO and RAO materials have more oxidized moieties compared to ASP, which can be reasonably attributed to the high degree of surface oxidation after the surface treatment of ASP with nitric acid and is consistent with FTIR results (*vide supra*).

These interpretations are reinforced by survey XPS data for ASP, AO, and RAO, which confirmed the presence of C, N, S, and O (Fig. S1†). Following oxidation of ASP, the oxygen content in AO increased significantly, and after subsequent reduction with hydrazine, there was a slight decrease in the oxygen content and an increase in the nitrogen amount. These results agree with the CHNS elemental analysis data (Table 1). The oxygen content (remaining mass) was 1.7, 20.4, and 15.6 wt% for ASP, AO, and RAO, respectively. The nitrogen content increased from 1.3 wt% in ASP to 5.0 wt% in AO (after oxidation with nitric acid) and to 7.5 wt% in RAO (after reduction with hydrazine). The sulfur content was in the range of 6–8 wt% throughout. Moreover, a decrease in H/C molar ratio and an increase in O/C molar ratio suggests the removal of aliphatic chains and an increase in aromaticity following oxidation of ASP.

**Table 1:** CHNS elemental analysis data of ASP, AO, and RAO

Sample	%C	%H	%S	%N	%O (by difference)	H/C	O/C
ASP	80.6	7.8	8.6	1.3	1.7	1.16	0.02
AO	62.8	5.4	6.4	5.0	20.4	1.04	0.24
RAO	64.7	5.8	6.4	7.5	15.6	1.07	0.18

**Fig. 4:**
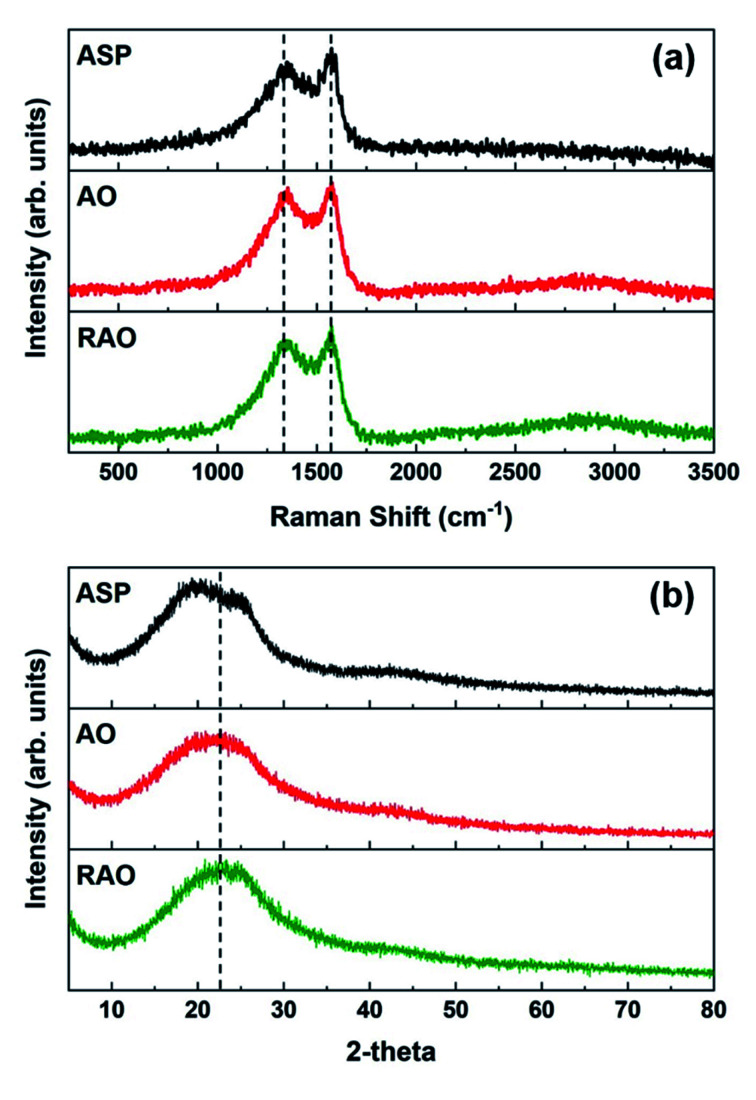
(a) Raman spectra and (b) XRD patterns of ASP, AO, and RAO.

The Raman spectra of ASP, AO, and RAO precursors showed two absorption bands at ∼1345 and ∼1575 cm^−1^, which are attributed to the D and G bands of graphene, respectively, further confirming the graphenic structure of these materials (Fig. 4a).^50^ The X-ray diffraction (XRD) patterns of all three precursor materials have a broad peak at 2*θ* of 20–25° indicating the poor long-range ordering of these graphene-like structures (Fig. 4b). The XRD pattern of ASP did show two features at 2*θ* angles of 20 and 25°, characteristic of the asphaltene γ and 002 peaks, respectively.^52^ After oxidation of ASP, the two asphaltene peaks disappeared, and we observed a broad XRD peak at around 22.5°, indicating the change in asphaltene structure and its conversion to oxidized carbon materials similar to what is observed in reduced graphene oxide.^53^

### 3.2. Graphene quantum dots from asphaltene and its derivatives

**Fig. 5:**
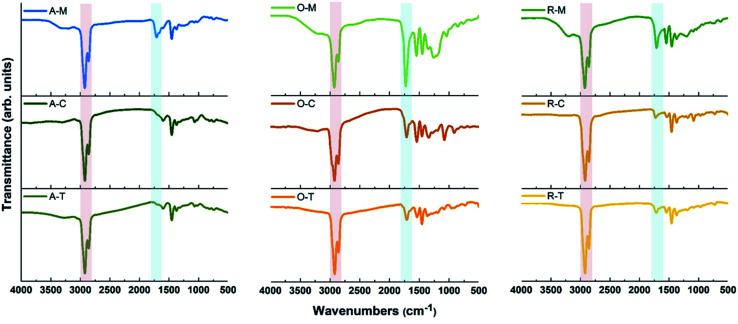
FTIR spectra of GQDs with indicated precursor–solvent combinations. The GQDs are labeled based on their respective precursor–solvent combinations (for example, A–M refers to the GQDs obtained from the ASP precursor dispersed in methanol).

Carbon precursors with different surface functionalities (*i.e.*, ASP, AO, and RAO) were dispersed in organic solvents with different polarity and hydrophobicity (*i.e.*, methanol, toluene, and chloroform) and sonicated for 3 hours to produce GQDs (Fig. 1). The resulting GQDs exhibited surface features consistent with their corresponding precursor material (Fig. 2 and 5). The choice of organic solvent also had a major impact on the surface chemistry of the GQDs. As shown in Fig. 5, the FTIR spectra of all GQDs show C–H and C=O stretching vibrations at 2850–2950 and 1700–1715 cm^−1^, respectively. If we compare the ratio of intensities of these two peaks in each spectrum, we observe that GQDs in methanol (A–M, O–M, and R–M) present stronger C=O stretching vibrations compared to their counterparts in chloroform and toluene, which implies that these GQDs have more oxygen-containing groups on their surface, and consequently they are more hydrophilic.

**Fig. 6:**
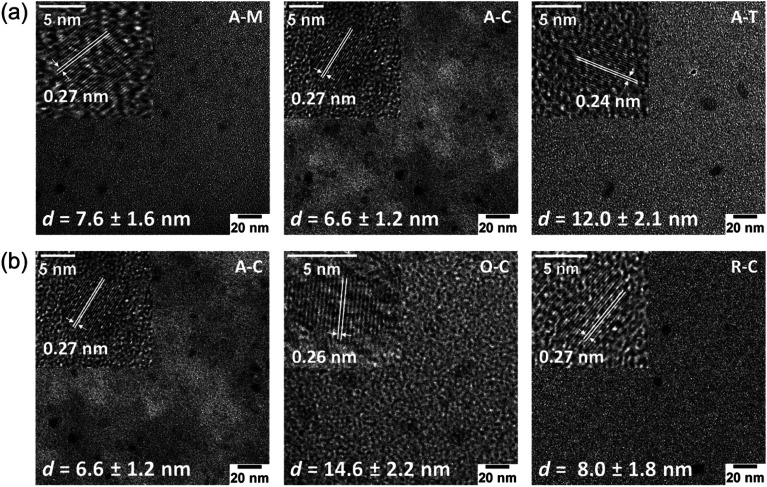
Bright-field TEM images of GQDs obtained from (a) asphaltenes in organic solvents and (b) asphaltene and its derivatives in chloroform. Inserts in TEM images show corresponding HRTEM images. The average diameters and standard deviation are also indicated for each sample. One sample is repeated because it appears in both sequences.

TEM images (Fig. 6) and their corresponding size distributions (Fig. S2†) reveal that these GQDs have lateral sizes below 20 nm. TEM images of GQDs obtained from asphaltene show average diameters of 7.6 ± 1.6, 6.6 ± 1.2, and 12.0 ± 2.1 nm for A–M, A–C, and A–T, respectively (Fig. 6a). Among these particles, A–T shows the larger TEM size, which could result from the high relative solubility of asphaltenes in toluene. This high solubility in toluene might not require the asphaltenes to dissociate into smaller particles during the ultrasonication process. We also investigated the average sizes of particles with different surface treatments (oxidized and reduced) suspended in chloroform (Fig. 6b). The A–C, O–C, and R–C particles show average diameters of 6.6 ± 1.2, 14.6 ± 2.2, and 8.0 ± 1.8 nm, respectively. The aforementioned changes in the H/C and O/C molar ratios (see Table 1) in the oxidized species indicate the removal of aliphatic chains, an increase in aromaticity, and formation of more oxygen-containing pendant groups.^54^ Therefore, we expect a larger aromatic core size for the oxidized particles, which is consistent with the larger TEM size observed for O–C.

HRTEM images (Fig. 6, insets) revealed the high crystallinity of GQDs and showed lattice fringe spacings of 0.24–0.27 nm that are slightly larger than the graphite (1120) lattice fringe (nominally 0.24 nm), which is attributed to the presence of larger heteroatoms (*e.g.*, sulfur) and functional groups that can slightly increase the interlayer spacing.^42^ Some GQDs also showed a lattice spacing of 0.35 nm, which corresponds to the graphite (002) lattice plane (Fig. S3†),^44^ suggesting that there is a stacking of at least a few graphene sheets, consistent with the AFM results shown below.

**Fig. 7:**
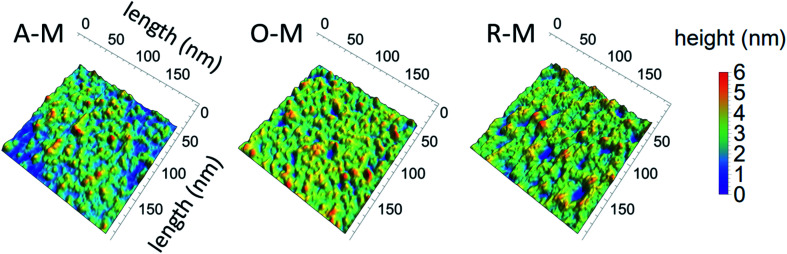
AFM images of GQDs and their corresponding height profiles for three representative samples (all in methanol).

AFM images of GQDs and their height profiles are given in Fig. 7 for samples suspended in methanol. Results for all samples are presented in Fig. S4.† AFM images of the GQDs in methanol (A–M, O–M, and R–M) show a smooth surface with a topographic height in the range of 0.35–1.28 nm, suggesting that most particles contain *ca.* 1–2 graphene layers.^33,36^ Among all GQD samples, A–M shows the smallest and narrowest height range (0.4 to 0.8 nm), suggesting that A–M mainly has a mono-layered structure. GQDs in chloroform (A–C, O–C, and R–C) exhibited a relatively rough surface with a topographic height in the range of 0.6 to 3.8 nm, indicating that most particles consist of *ca.* 1–7 graphene layers. Sample A–T yielded a greater height range (1.52–4.71 nm), suggesting more graphene layers (*ca.* 3–9) for this sample; this is consistent with the TEM results and might be due to the smaller degree of fractionation of asphaltene in toluene during the synthesis.

**Fig. 8:**
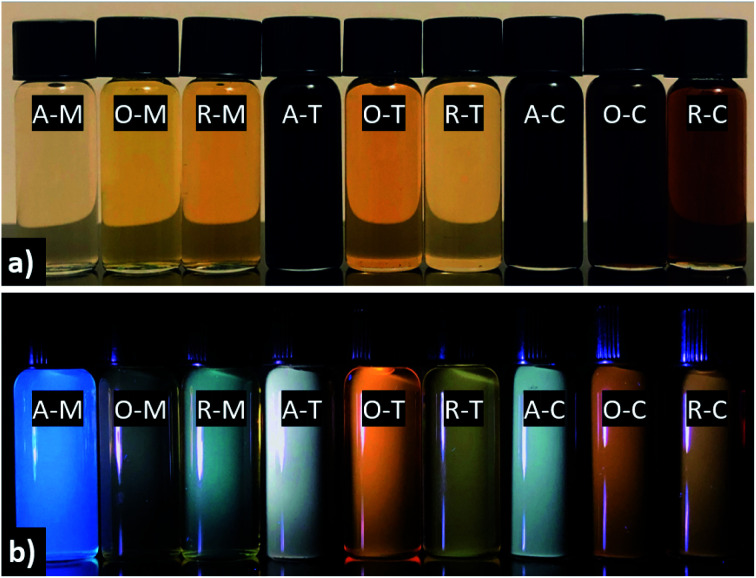
Photographs of GQDs with indicated precursor–solvent combinations under (a) ambient and (b) UV light. The concentration of all samples is ∼1 mg mL^−1^. Photographs were taken with an iPhone X camera.

The visual appearance of GQDs obtained from different precursor–solvent combinations upon exposure to ambient and UV light (*i.e.*, 365 nm) is distinctive, with colors ranging from light yellow to black under visible light and fluorescence colors from blue to green, yellow, orange, or white under UV light (Fig. 8). The darker color of A–T, A–C, O–C, and R–C might be due to their multi-layered structure (*ca.* >5 graphene layers) shown by AFM analysis. Among all samples, GQDs in methanol tend to give the “bluest” fluorescence, whereas in toluene or chloroform one finds principally yellow, orange, or white emission. This is explained by the nearly mono-layered structure of GQDs in methanol, which contributes to their smaller particle size compared to their counterparts in toluene or chloroform, and consequently blueshifted PL.

**Fig. 9:**
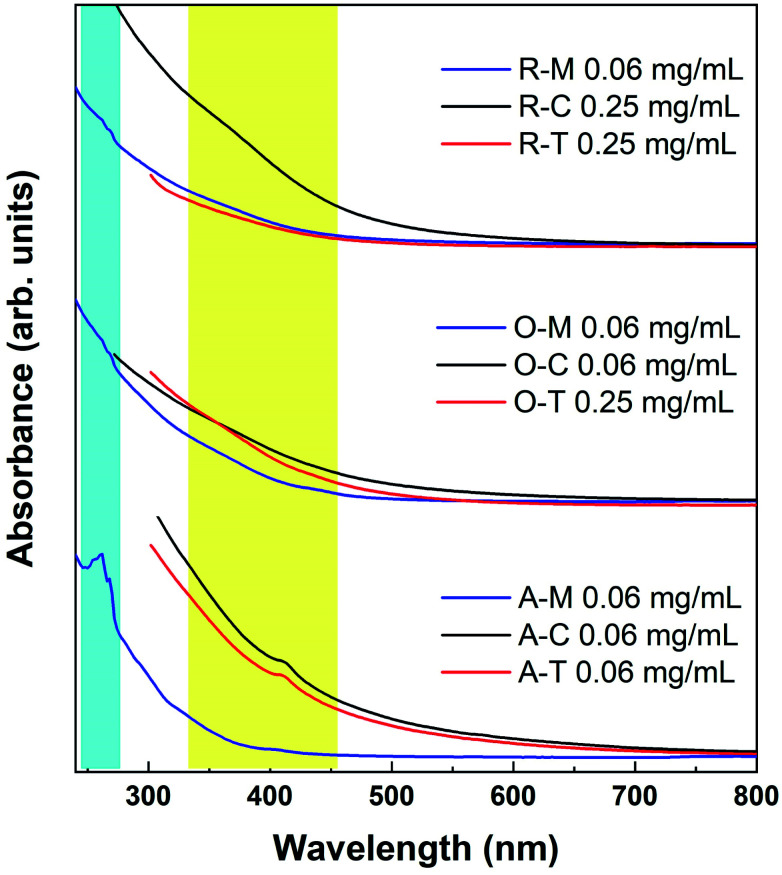
Absorption spectra of GQDs in methanol (blue lines), toluene (red lines), and chloroform (black lines). The lines are intentionally cut off at the solvent absorption onsets.

The UV-Vis spectra show a smoothly increasing absorption as a function of decreasing wavelength for GQDs in all three solvents (Fig. 9). A shoulder at *ca.* 310 nm is believed to correspond to an n–π* transition related to nitrogen or oxygen-containing groups, while a peak at 230–270 nm is believed to correspond to a π–π* transition of aromatic sp^2^ domains.^55^ However, the apparent features are invariably weak in the UV-Vis spectra, which can be attributed to the presence of the organic solvent, a wide size distribution, or a variety of surface states. We observed similar features in some cases where weak absorptions were resolvable around 400 and 270 nm in some samples (yellow and blue shaded regions in Fig. 9). Because there appears to be a precursor/solvent dependence on their strength (this was checked in multiple repeat measurements), we believe that these traits are most likely related to the specific surface states.

The absorption tail in the visible region (starting from 400 nm and extending toward longer wavelengths) are the result of surface states associated with nitrogen, sulfur, or oxygen-containing groups and the degree of conjugation in the graphene skeleton.^55^ Therefore, darker-colored samples in Fig. 8 (*e.g.*, A–T) likely consist of a wide distribution of fractions with a high content of nitrogen-, sulfur-, and oxygen-based groups and a larger variation of aromatic sp^2^ sites that may be conjugated with edge functional groups. In comparison to A–T, A–M is almost transparent under ambient light (see Fig. 8). This implies that A–M likely contains fractions with smaller aromatic sites and fewer N, S, and O functionalities, which results from the low solubility of asphaltenes in methanol. In other words, the visual appearance of GQDs also depends on the degree of solubility of the precursor material in the chosen solvent, which is in turn governed by the surface chemistry.

**Fig. 10:**
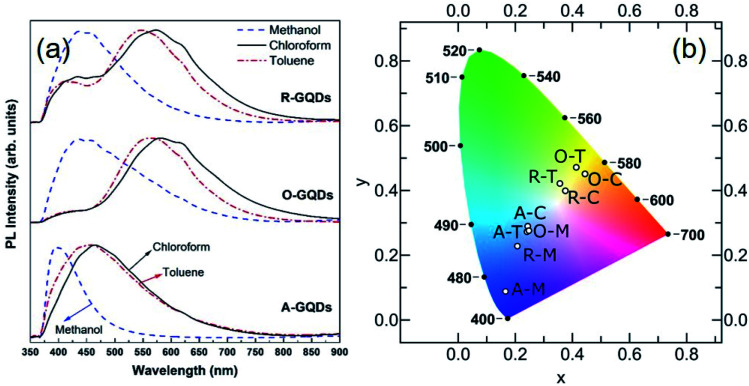
(a) Photoluminescence spectra of GQDs dispersed in methanol, chloroform, or toluene. (b) CIE diagram of GQDs: CIE diagram shows the CIE color coordinates calculated from the spectra, which agrees well with the colors observed under the UV light.

The PL spectra of the GQDs (Fig. 10a) show the range of emission wavelengths, which sweep out a trace from blue through white to pale orange on the CIE color diagram (Fig. 10b). GQDs obtained from asphaltene show a PL spectrum with maxima in the blue region at 390, 445, and 465 nm for A–M, A–C, and A–T, respectively. After oxidation, the PL spectra broadened and shifted to blue (440 nm) for O–M and all the way into green (565 nm) for O–T and yellow (585 nm) for O–C because of the formation of electron-withdrawing groups on the GQD surface.^55,56^ After the subsequent reduction, there was a little change for GQDs in methanol, which retained a strong blue PL peak at 440 nm; whereas, for the other two solvents the green–yellow emissions remained, but a new, weaker peak developed at 440 nm, suggesting a mixture of at least two surface states consisting of the blue emission and the green–yellow emission.

Broadly speaking, PL mechanisms of GQDs can be related to the quantum confinement and/or surface effects. The aromatic sp^2^ domain size is known to control quantum size effects with the graphenic core being attributed to the main blue PL center for GQDs.^57^ A greater degree of surface oxidation usually redshifts the PL emission of GQDs because functional groups such as carboxyl and epoxy groups on the surface and edges of GQDs usually induce the formation of surface oxidation states with a series of emissive traps.^57,58^ We observed that GQDs obtained from asphaltene show a blue PL compared to their oxidized and reduced counterparts (see Fig. 10a) because of having more electron-donating (ED) groups such as –OH and –NH on their surface. These groups are believed to enrich the electron density of the π-conjugated structure and blueshift the PL.^55,56^ Oxidation of asphaltene results in GQDs with more electron-withdrawing (EW) groups such as carboxyl and amide and causes a PL redshift.

The fluorescence decays were non-exponential, with only A–M showing somewhat close to a mono-exponential decay (Fig. S5†). If the lifetime is related to the particle size or broadening effects, then one should expect the decay to be characterized by a distribution of lifetimes; whereas if the decays are related to two or more (separate) populations, then the lifetime distribution would resolve into a simple combination of exponentials. Single- and bi-exponential models yielded relatively poor fits and left significant patterns in the residuals. We believe that, instead, there is a continuous distribution of lifetimes present in all samples, reflecting the dispersion of sizes and surface states. Since there is no way to know *a priori* what this distribution is, and because the mean lifetimes can be highly dependent on the chosen model,^59^ a characteristic decay time was instead calculated by integration (*i.e.*, by numerically finding the midpoint of the area under the decay curves), leading to decay times ranging from about 1.6 to 2.5 ns in all samples except A–M, which had the longest time at 3.7 ns (this is also the sample with a distinctly different emission spectrum from all the others, with a maximum in the UV region).

**Fig. 11:**
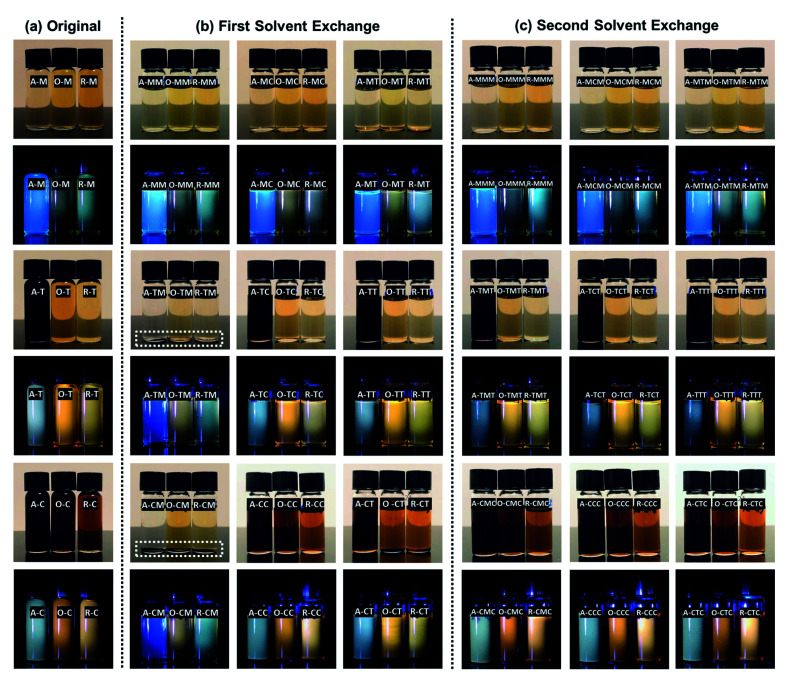
A library of GQDs (a) original samples, (b) after the first solvent exchange, and (c) after the second solvent exchange under ambient and UV light. The concentration of all samples is ∼1 mg mL^−1^. The labeling system represents the precursor and the solvents; for example, A–MT represents A–M nanoparticles that were dried and re-dispersed in toluene. Photographs were taken with an iPhone X camera.

To investigate the solvent effect on GQD optical properties, each GQD solution was added in equal parts into three glass containers, air-dried, and re-dispersed in methanol, chloroform, or toluene. Images of the GQDs after this first solvent exchange, under both ambient and UV light, showed that the role of the solvent on the optical response of the GQDs is critical (Fig. 11b). For example, R–T exhibits a yellow PL upon exposure to UV light but when R–T nanoparticles are dried and re-dispersed in methanol (labeled R–TM), they show a greenish-white PL instead. This indicates that the GQDs obtained from different precursor–solvent combinations can be further selected by the choice of organic solvents with different polarity and hydrophobicity during a post-synthesis step. As a further examples, see GQD samples in toluene after redispersion in methanol, chloroform, and toluene (Fig. 11b).

The GQDs were re-dispersible in the same or the other two solvents after drying, but the extent of dispersibility depended both on the surface chemistry of the GQDs and the solvent properties such as polarity and hydrophobicity. For example, A–C nanoparticles were only partially re-dispersible in methanol (see A–CM in Fig. 11b; a precipitate is visible under ambient light), but they were completely re-dispersible in chloroform and toluene (see Fig. 11b, A–CC and A–CT). To test whether the optical changes were due to the solvent or selective GQD redispersion, all samples were re-dispersed once more, this time back into their original solvent (a second solvent exchange; Fig. 11c). We observed that the particles were completely dispersible in their original solvents, and their original response was fully retrievable.

The PL spectra of the GQDs after the first and second solvent exchanges are shown in Fig. S6 and S7.† The solvent-related spectral shifts are likely caused by a selection effect in which only particles with a certain character (*i.e.*, size or surface chemistry) are fully re-dispersed after the solvent exchange. Asphaltene oxidation with HNO_3_ is known to result in water-soluble and water-insoluble fractions, with the more water-soluble fraction being more aliphatic, and the more water-insoluble fraction consisting of a more aromatic portion that is more resistant to oxidation and higher in molecular weight.^60^ The PL spectra of the solvent-exchanged samples also show that the re-dispersion of O and R samples from chloroform or toluene into methanol resulted in a dissolved fraction showing a PL spectrum similar to that of original methanol samples, which further supports the selection effect. Physical agglomeration of GQDs could also play a role in the emission spectra; however, there were no spectral shifts observed by varying sample concentrations over a ∼100-fold range from 0.03 mg mL^−1^ to 2.5 mg mL^−1^ (Fig. S8†). Although positive solvatochromism has been reported in GQDs previously,^61–64^ it is unlikely to be responsible for the spectral shifts observed here because there was no consistent trend as a function of solvent polarization index or static dielectric constant.

Solvent exchange investigations showed that the re-dispersion of asphaltene and its oxidized and reduced derivatives from chloroform or toluene into methanol (see the selected area in Fig. 11b) results in a precipitate and a dissolved fraction, showing a blue PL similar to that of original methanol samples (*i.e.*, a selection effect). This implies that asphaltene and its derivatives contain two fractions, one of them being soluble in all three solvents, and the second one being soluble only in chloroform and toluene. This could explain why GQDs in methanol do not show a double-peaked PL spectrum. The features observed in the PL spectra of oxidized and reduced samples dispersed in chloroform and toluene (O–C, R–C, O–T, and R–T) can be explained also by the combination of more hydrophilic and more hydrophobic fractions present in the precursor materials. Thus, asphaltene and its derivatives contain both hydrophobic and hydrophilic fractions, and these fractions can be selected by dispersing the GQDs in solvents with different polarity and hydrophobicity. Moreover, the PL quantum yield (QY) of GQD in methanol was in the range of 11 to 16%, close to the values calculated for water-soluble GQDs synthesized from asphaltene.^37^ GQDs in toluene and chloroform had a lower QY, probably due to the presence of both hydrophilic and hydrophobic fractions. Work is in progress to further improve the QY of these asphaltene-derived GQDs.

**Fig. 12:**
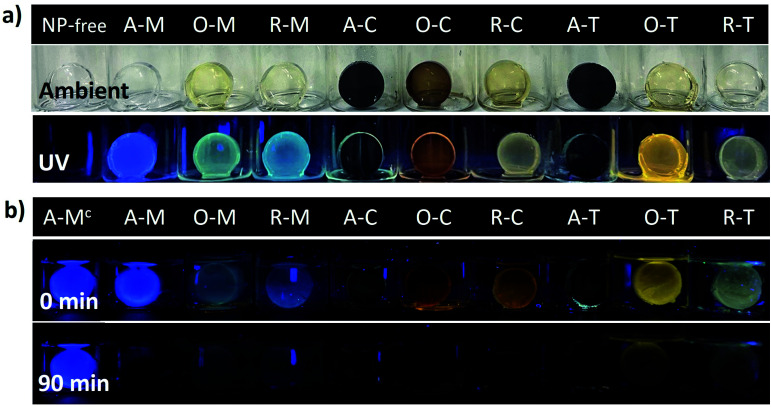
(a) Photographs of GQDs incorporated into silica matrices under ambient and UV light [NP-free refers to nanoparticle-free silica matrices]. (b) GQD-silica hybrid materials immediately after initial exposure to aqueous solution of 4-nitrophenol (1 μM) and after 90 minutes. A–M^c^ refers to a control sample without the analyte (*i.e.*, water only), showing no quenching over the 90 minutes time frame.

Many future applications (*e.g.*, sensors, light emitting diodes, *etc.*) would benefit from the GQDs being incorporated into different host materials and being chemically accessible. To demonstrate this concept, we finally incorporated the GQDs into silica gel matrices. Fig. 12a shows the visual appearance and the PL of the GQD-silica hybrid materials before and after exposure to 365 nm light. In all cases, the gels were clear and transparent in ambient lighting and, under a UV lamp, they showed visual emission colors consistent with those of the original GQDs (compare Fig. 8 and 12a). Thus, asphaltene-derived GQDs can be selected according to the solvent dispersion methods and then applied to the formation of GQD-hybrid materials that preserve their solvent-selected luminescent properties.

Finally, one potential application of the GQD-silica hybrids is briefly demonstrated. Exposure of GQDs to nitro-aromatic compounds (NACs) is known to quench their PL, a feature which has been widely investigated for NAC-sensing applications.^65–72^Fig. 12b qualitatively shows the quenching of the PL from the GQD-silica gel hybrids upon exposure to an aqueous solution of 4-nitrophenol (1 μM). This confirms that the GQDs are readily accessible even after incorporation into the silica host material. Most of the samples were fully quenched over a period of 90 minutes, with the fastest quenching for the chloroform-derived hybrids and the slowest for the toluene-derived ones. This initial demonstration shows that the solvent effects described above can be employed to optimize the fluorescence character of GQD-hybrid sensor materials. This initial demonstration could lead to novel liquid or vapor sensors for NACs or other analytes of interest, in which the GQD properties can be first carefully tuned *via* solvent exchange and then utilized toward the development of more complicated hybrid sensing structures.

## 4. Conclusions

We synthesized N, S, and O-functionalized GQDs with tunable PL and hydrophobicity from asphaltene and its oxidized and reduced derivatives *via* a facile and scalable ultrasonication method. Using asphaltene as a precursor material has several advantages, including (1) it is an abundant and low-cost carbon source, (2) it naturally contains N and S heteroatoms to modify GQD properties, and (3) its chemical structure is like the GQD core structure, which facilitates GQD synthesis. Tuning the surface chemistry of asphaltene through sequential oxidation and reduction permitted the production of GQDs with tunable properties by incorporating more oxygen and nitrogen-containing functional groups, as confirmed by spectroscopic characterization. TEM and AFM imaging revealed that asphaltene-derived GQDs have mono-layered or multi-layered structures with lateral sizes in the range of ∼5–15 nm. Optical and solvent exchange studies confirmed that, by changing the precursor surface chemistry and the solvent in which the GQDs were synthesized, it is possible to tune their hydrophobicity and optical properties to have luminescence colors ranging from blue to yellow/orange or pale white. PL quenching of GQDs incorporated into silica gels materials upon exposure to NACs confirms the chemical accessibility of the GQD within the gel matrices, implying that more advanced applications could readily be developed from solvent-selected GQD hybrid materials.

## Conflicts of interest

The authors declare that they have no known competing financial interests or personal relationships that could have appeared to influence the work reported in this paper.

## Supplementary Material

NA-004-D2NA00445C-s001
