# Supplementary material for: Synthesis and properties of multi-functionalized graphene quantum dots with tunable photoluminescence and hydrophobicity from asphaltene and its oxidized and reduced derivatives
Source: Nanoscale Adv. 2022 Aug 22;4(19):4080–93. doi: 10.1039/d2na00445c (PMC9514569; doi:10.1039/d2na00445c)
Supplement: NA-004-D2NA00445C-s001 [file NA-004-D2NA00445C-s001.pdf]

Electronic Supplementary Material (ESI) for Nanoscale Advances.
This journal is © The Royal Society of Chemistry 2022

Supporting Information for

Synthesis and Properties of Multi-Functionalized Graphene Quantum Dots with Tunable Photoluminescence and Hydrophobicity from Asphaltene and Its Oxidized and Reduced Derivatives

Maryam Aghajamali[a,*], Mariana Arpini Vieira[b,c], Razieh Firouzi-haji[b], Kai Cui[c], Jae-Young Cho[c], Adam Johan Bergren[c,d,*], Hassan Hassanzadeh[a,*], and Alkiviathes Meldrum[b,*]

[a] Department of Chemical & Petroleum Engineering, Schulich School of Engineering, University of Calgary, Calgary, AB T2N 1N4, Canada

[b] Department of Physics, University of Alberta, Edmonton, AB T6G 2E1, Canada

[c] Nanotechnology Research Centre, National Research Council of Canada, Edmonton, AB T6G 2M9, Canada

[d] Department of Chemistry, University of British Columbia, Kelowna, BC V1V 1V7, Canada

***Corresponding authors:** M. Aghajamali (maryam.aghajamali@ucalgary.ca); A. J. Bergren (adam.bergren@nrc.ca); H. Hassanzadeh (hhassanz@ucalgary.ca); A. Meldrum (ameldrum@ualberta.ca).

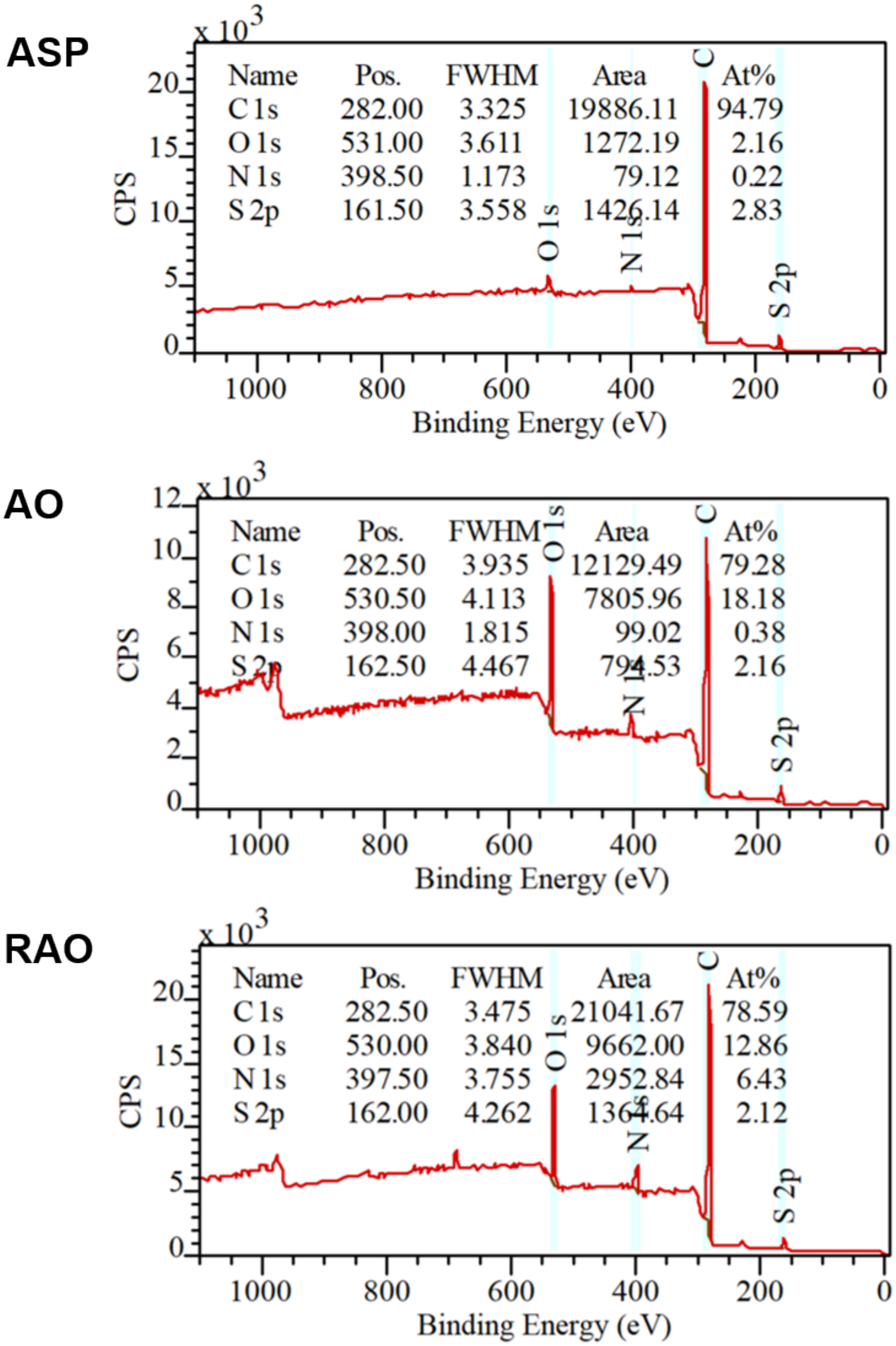

Figure S1. Survey XPS of asphaltene (ASP), asphaltene oxide (AO), and reduced asphaltene oxide (RAO).

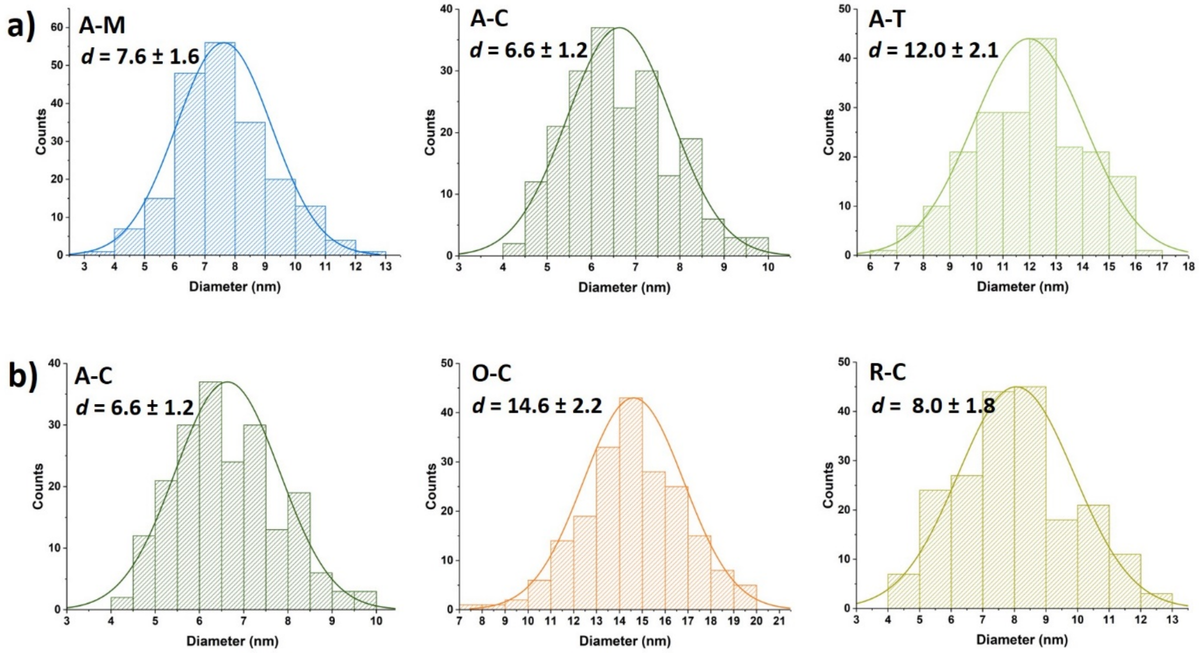

Figure S2. GQD size distributions obtained from TEM images by counting 200 particles.

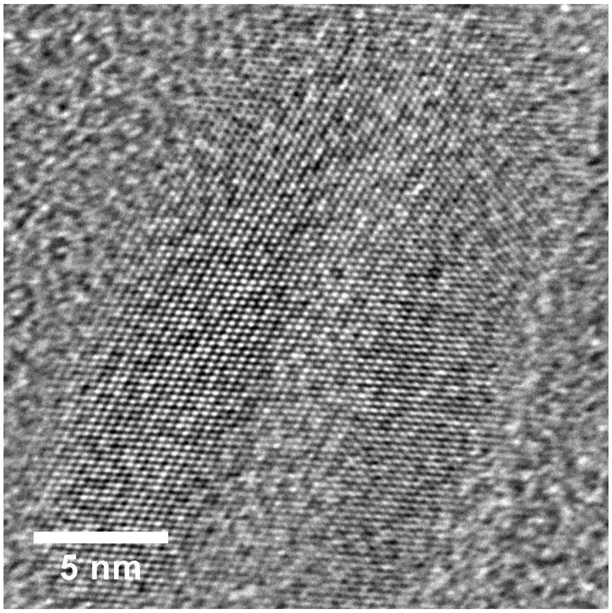

Figure S3. Representative HRTEM image of R-C with the lattice spacing of 0.35 nm characteristic of the graphite (002) lattice fringe.

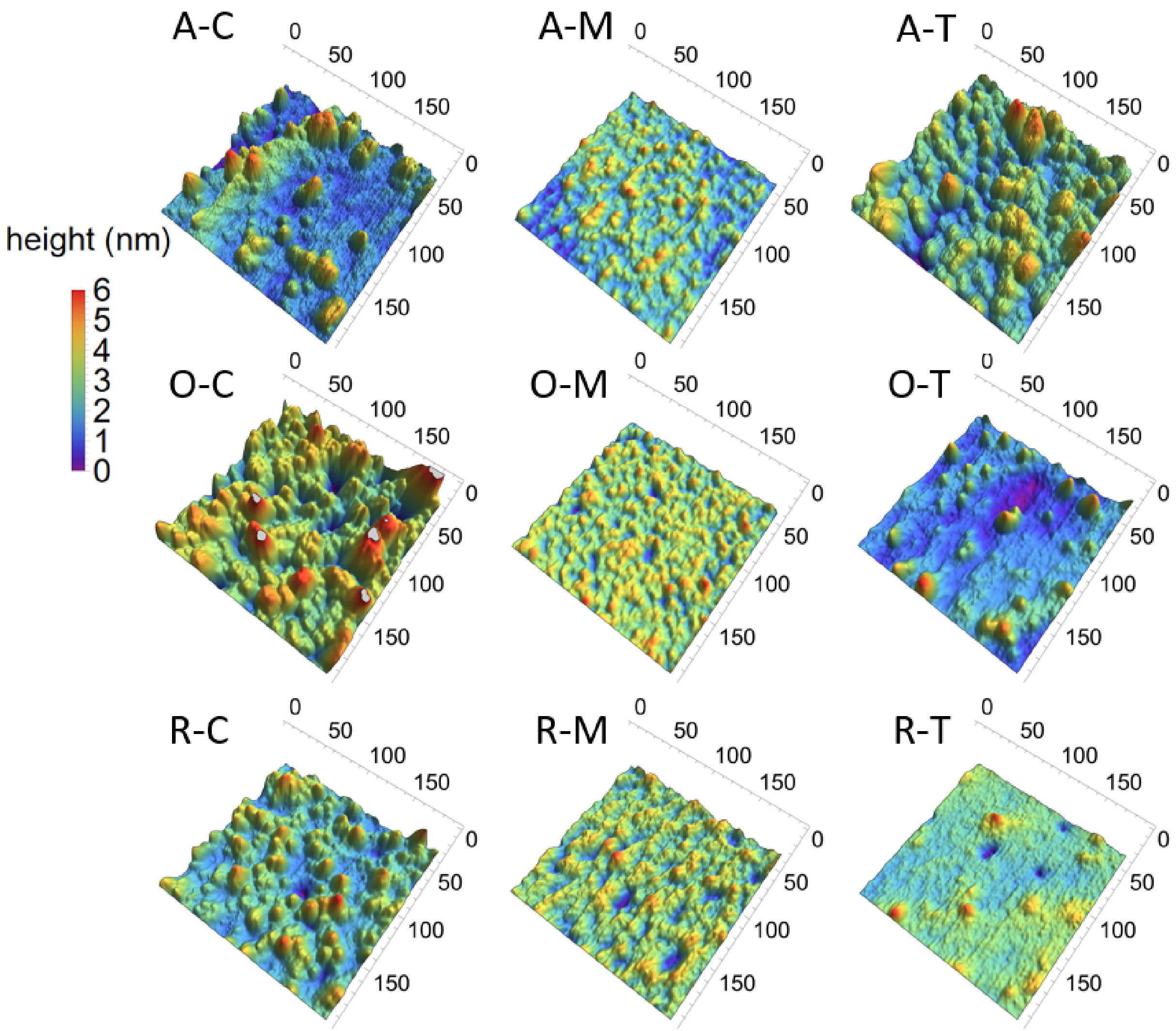

Figure S4. (a) AFM surface plots showing different GQDs.

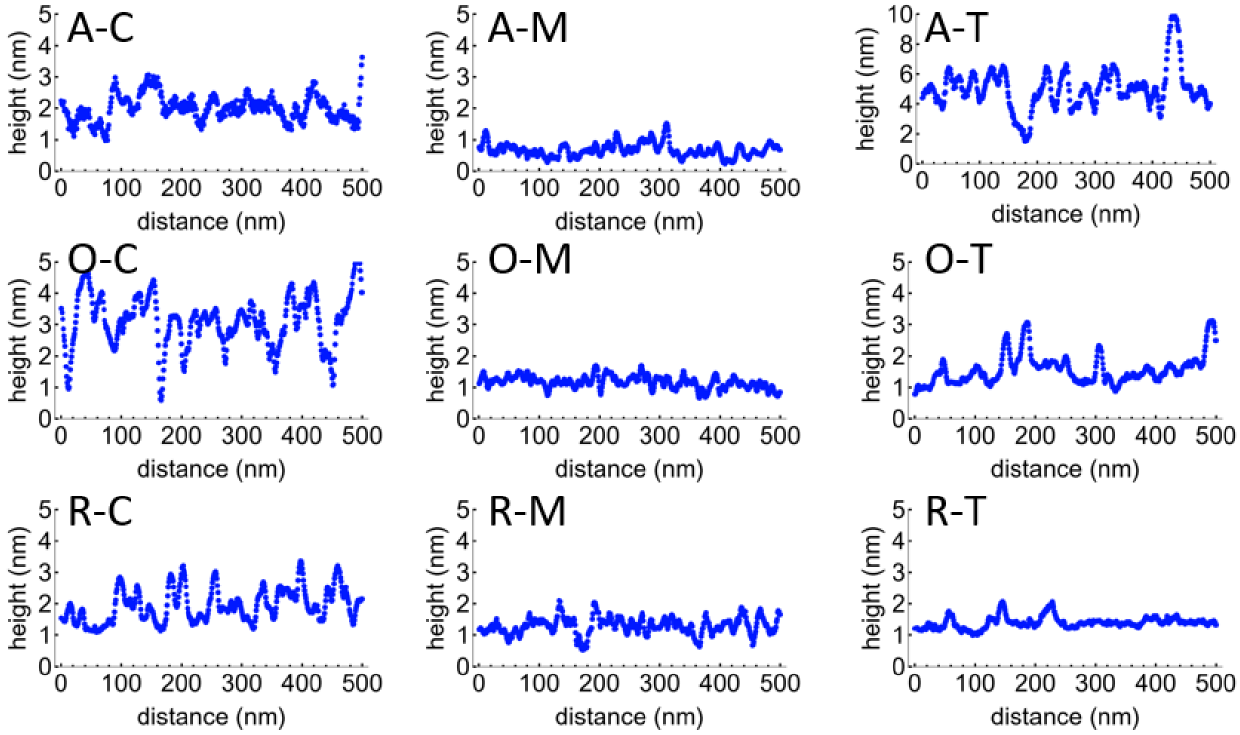

Figure S4. (b) AFM line profiles (selected randomly) corresponding to the images in Fig. S4a.

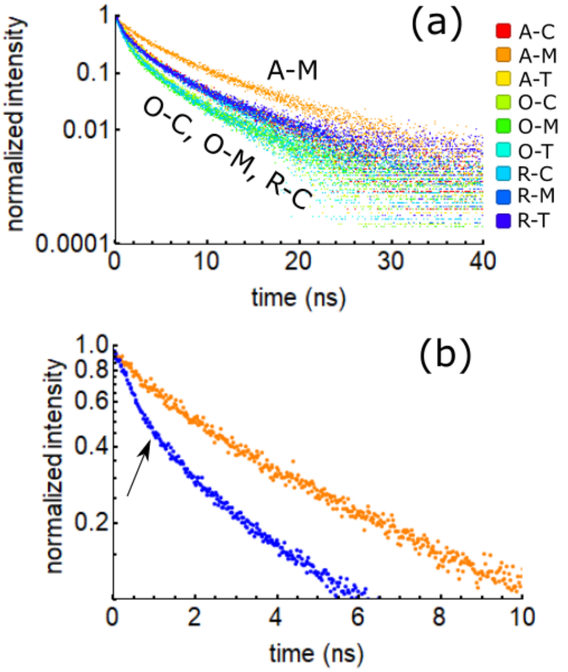

Figure S5. (a) Luminescence decays for all GQD samples. A-M has the longest lifetime and O-C, O-M, and R-C have the shortest. The other 5 samples are clustered in between the labeled curves but are very close to the latter three. (b) compares A-M with R-M, where we can see the change in slope of the R-M decay denoted by the arrow.

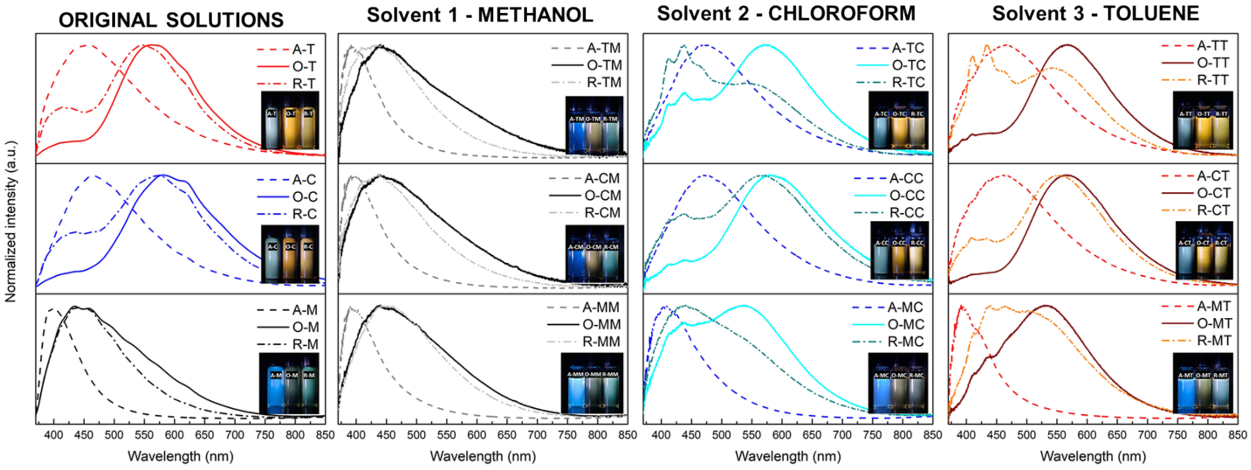

Figure S6. Photoluminescence spectra of GQDs in original solvents and after the first solvent exchange. The concentration of all samples is ~1 mg/mL.

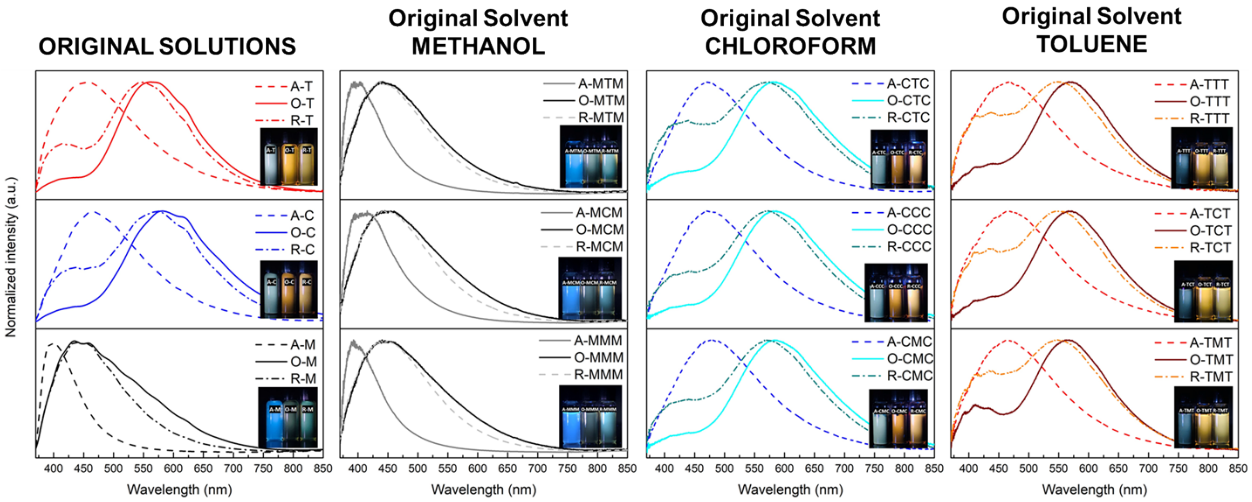

Figure S7. Photoluminescence spectra of GQDs in original solvents and after the second solvent exchange. The concentration of all samples is ~1 mg/mL.

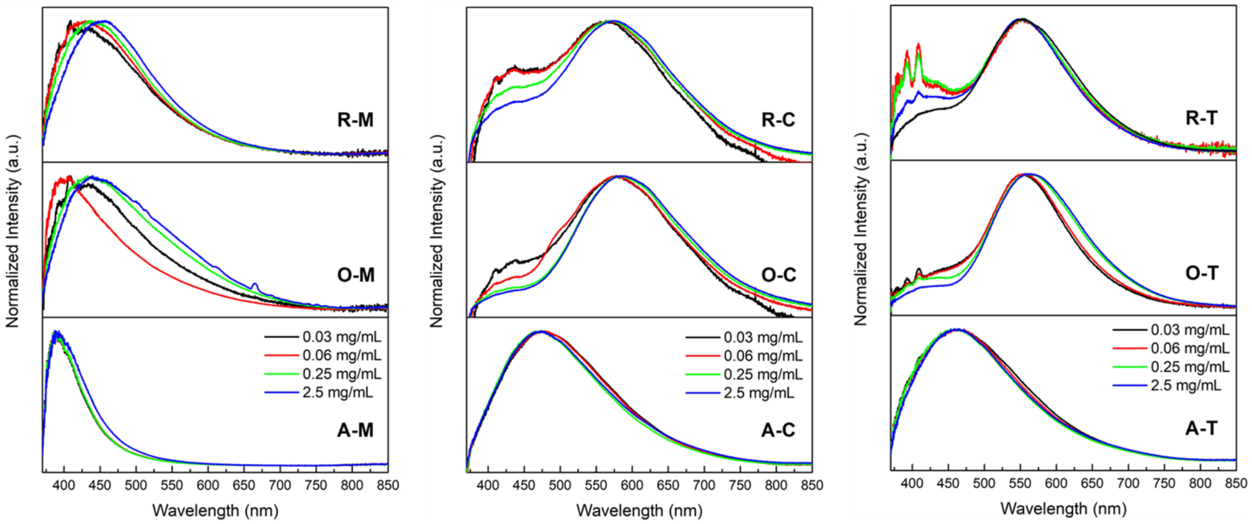

Figure S8. Concentration-dependent photoluminescence spectra of GQDs.
